# 2109: Interleukin 4-induced protein 1 as a biomarker and treatment option in multiple sclerosis

**DOI:** 10.1017/cts.2017.21

**Published:** 2018-05-10

**Authors:** Stephanie Davis, Jeffrey Huang

**Affiliations:** Georgetown - Howard Universities, Washington, DC, USA

## Abstract

OBJECTIVES/SPECIFIC AIMS: The overall objective of this proposal is to establish and modulate the inflammatory profile of individuals across the spectrum of multiple sclerosis (MS), with a focus on determining the potential of interleukin 4-induced protein 1 (IL4I1) as a possible marker of progression and modulator of inflammation in human blood samples. METHODS/STUDY POPULATION: The proposed experimental approach involves isolating plasma and peripheral blood mononuclear cells (PBMCs) from individuals across the spectrum of MS phenotypes, and analyzing these samples primarily by quantitative polymerase chain reaction (qPCR) and enzyme-linked immunosorbent assay (ELISA) methods. Specifically, study groups include: (1) actively relapsing-remitting MS (a-RRMS), (2) non-actively relapsing-remitting MS (n-RRMS), (3) non-active secondary-progressive MS (SPMS), (4) other autoimmune diseases (OAD), (5) healthy controls (HC). RESULTS/ANTICIPATED RESULTS: We expect that IL4I1 treatment increases regulatory cytokine (eg, IL10, TGFb) expression while decreasing Th1 and Th17-derived cytokines (IFNg, IL17), as well as increasing relative composition of regulatory cells (Th2, Treg, M2) as compared with Th1, Th17, M1 (aim 1). Preliminary data on healthy control cells support this prediction. Our central hypothesis is that IL4I1 level indicates the body’s ability to repair itself. As such, we anticipate that all MS groups are deficient in IL4I1, to varying degrees, such that HC>n-RRMS>a-RRMS>SPMS. HC have full repair capacity. RRMS>SPMS as remission indicates existent repair capacity, which is lost in SPMS. n-RRMS>a-RRMS since both, as RRMS, capable of repair response, but a-RRMS triggered this response more recently in response to more recent relapse. In all groups, we expect IL4I-treatment to mitigate inflammation (aim 2). Finally, we expect that H_2_O_2_ production by IL4I1 is a key player in IL4I1 function, and that H2O2 will preferentially induce oxidative stress to pro-inflammatory subsets of PBCMs (aim 3). DISCUSSION/SIGNIFICANCE OF IMPACT: MS is a chronic inflammatory neurodegenerative disease of the central nervous system that, with an average age of onset of 34, afflicts over 2.3 million individuals worldwide during many of the most productive years of their lives. The pathogenesis of MS, which involves autoimmune destruction of myelin, is poorly understood. Accurate biomarkers, which could predict disease progression, are yet to be identified and would provide valuable information to patients and their treating clinicians. Likewise, effective treatments are few and in high demand. IL4I1 is a promising candidate for both roles.

